# The association between indoor temperature and body mass index in children: the PIAMA birth cohort study

**DOI:** 10.1186/1471-2458-13-1119

**Published:** 2013-12-05

**Authors:** Floor R Scheffers, Marga BM Bekkers, Marjan Kerkhof, Ulrike Gehring, Gerard H Koppelman, Maarten Schipper, Annemien Haveman-Nies, Alet H Wijga

**Affiliations:** 1 Department of Human Nutrition, Wageningen University, Wageningen, the Netherlands; 2 National Institute for Public Health and the Environment (RIVM), Centre for Nutrition, Prevention and Health Services (pb 101), PO Box 1, Bilthoven 3720, BA, the Netherlands; 3 Department of Epidemiology and Bioinformatics, University of Groningen, University Medical Center Groningen, Groningen, the Netherlands; 4 Institute for Risk Assessment Sciences, Utrecht University, Utrecht, the Netherlands; 5 Department of Pediatric Pulmonology and Pediatric Allergology, University of Groningen, University Medical Center Groningen, Beatrix Children’s Hospital, GRIAC Research Institute, Groningen, the Netherlands; 6 Department of Statistics Mathematical Modelling and Data Logistics, National Institute for Public Health and the Environment, Bilthoven, the Netherlands

**Keywords:** Body mass index, Children, Ambient temperature, Indoor environment, Overweight, Energy balance

## Abstract

**Background:**

Several experimental studies showed consistent evidence for decreased energy expenditure at higher ambient temperatures. Based on this, an association between thermal exposure and body weight may be expected. However, the effect of thermal exposure on body weight has hardly been studied. Therefore, this study investigated the association between indoor temperature and body mass index (BMI) in children in real life.

**Methods:**

This longitudinal observational study included 3 963 children from the Dutch Prevention and Incidence of Asthma and Mite Allergy (PIAMA) birth cohort that started in 1996. These children were followed from birth until the age of 11 years. Winter indoor temperature (living room and bedroom) was reported at baseline and BMI z-scores were available at 10 consecutive ages. Missing data were multiply imputed. Associations between indoor temperature and BMI were analyzed using generalized estimating equations (GEE), adjusted for confounders and stratified by gender. In a subgroup of 104 children, bedroom temperature was also measured with data loggers.

**Results:**

Mean reported living room and bedroom temperature were 20.3°C and 17.4°C, respectively. Reported and measured bedroom temperatures were positively correlated (*r* = 0.42, *p* = 0.001).

Neither reported living room temperature (-0.03 ≤ β ≥ 0.04) and bedroom temperature (-0.01 ≤ β ≥ 0.02) nor measured bedroom temperature (-0.04 ≤ β ≥ 0.05) were associated with BMI z-score between the age of 3 months and 11 years.

**Conclusions:**

This study in children did not support the hypothesized association between indoor temperature and BMI in a real life setting.

## Background

Both on theoretical grounds and from experimental studies it is well established that at lower ambient temperatures energy expenditure increases to maintain body temperature [1]. This implies that – all other things being equal – less energy is available for storage in the form of fat at low ambient temperatures than at higher temperatures. Based on such considerations and on the observation that in the USA and the UK indoor temperatures have increased over the past decades, Johnson et al. proposed the hypothesis that an increase in indoor temperatures may have been one of the factors that contributed to the current obesity epidemic [1]. In the UK the mean living room temperature increased from 18.3°C in 1978 to 19.1°C in 1996 and the mean temperature in bedrooms increased from 15.2°C in 1978 to 18.5°C in 1996. Johnson et al. suggest that these changes might have been sufficient to affect energy balance and might thus have contributed to the increase in the prevalence of overweight and obesity.

However, studies on the association between ambient temperature and energy balance or adiposity in real life conditions are scarce and there is currently insufficient evidence for a role of ambient temperature in the development of overweight. A recent study conducted in the USA investigated geographical associations between obesity prevalence and mean annual outdoor temperatures, altitude and urbanization [2] . They observed an approximately parabolic relationship between mean annual temperature and obesity prevalence with the lowest prevalence of obesity in counties with mean annual temperatures of 0.0-4.9°C. This association was no longer statistically significant after adjustment for altitude. In line with Johnson’s hypothesis, an Italian study observed a twofold increased risk for incident obesity during 6 years of follow-up in adults having indoor temperatures higher than 20°C compared to those having indoor temperatures lower than or equal to 18°C [3].

The aim of our study was to further elucidate the association between ambient temperature and adiposity. Therefore, we investigated, in a real life setting, the association between indoor temperature (living room and bedroom) and body mass index (BMI) in a cohort of children followed from birth up to the age of 11 years.

## Methods

### Study design and study population

The Prevention and Incidence of Asthma and Mite Allergy (PIAMA) study is a birth cohort study in the Netherlands that started in March 1996. During their pregnancy 7 862 mothers were recruited from the general population in three different regions of the Netherlands. The recruitment of the mothers started during their first visit to a prenatal health clinic with the help of 52 midwifes. No exclusion criteria were used. In total 4 146 mothers agreed to participate and gave written informed consent. All children were born in 1996 or 1997. There were 183 mothers lost to follow-up during the first data collection on the child. For this reason the baseline study population consisted of 3 963 children. The first questionnaire was completed by the parents during pregnancy. The second questionnaire was completed by the parents when the child was 3 months old, and subsequently, every year until the child was 8 years old. In 2008, at the child’s age of 11 years, the parents received a questionnaire again. The study protocol was approved by the Medical Ethics Committees of the participating institutes (Rotterdam, MEC 132.636/1994/39 and 137.326/1994/130; Groningen, MEC 94/08/92; Utrecht, MEC-TNO 95/50). Detailed descriptions of the study design has been published previously [4,5]. For our analyses, children were excluded if data were missing on all BMI measurements (n = 32), bedroom and living room temperature (n = 89), or confounders (n = 497). Furthermore, children with reported values for living room temperature lower than 10°C were also excluded (n = 2). After exclusion, data from 3 343 children were available for the analyses of the original data.

### BMI

Child’s height (in cm) and weight (in kg) were reported by the parents at the age of 3 months, 1 year, 2 years, 3 years, 4 years, 5 years, 6 years, 7 years, 8 years and 11 years. Parents were asked to report the child’s weight and height measured by a medical professional, if this measurement was within the last 3 months. Otherwise parents were asked to measure weight and height of the child themselves without shoes and heavy clothes. Based on data on child’s height and weight the continuous outcome variable BMI z-score was calculated. First, BMI was calculated as weight divided by height squared in meters (kg/m^2^). BMI was not calculated if data on weight or height were missing and/or if the period between the measurements of weight and height was longer than 60 days. Subsequently, gender and age-specific BMI z-scores were calculated by using the reference growth curves of the Dutch fourth nationwide growth study that was performed in 1997 [6].

### Indoor temperature

Winter indoor temperature in both living room and bedroom was reported by the parents in the first year of life. The following open question on temperature was asked for living room and bedroom: “At what temperature do you keep your room in winter?” If people did not use the heating, they could choose the additional answer “heating is not used”. Both living room and bedroom temperature were divided into two groups: “heating used” versus “heating not used”. Temperatures within the category “heating used” were used as a continuous variable.

In a subgroup of children (n = 104) bedroom temperature was measured with data loggers every 30 minutes during a period of approximately 2 weeks. These measurements took place in November, December, January and February of the years 1997, 1998 and 1999 and were used to calculate a mean measured bedroom temperature for each child in this subgroup.

### Statistical analyses

Data were analyzed using SAS 9.2 (SAS Institute, Inc., Cary, NC, USA). Descriptive statistics were used to describe the characteristics of the study population. The regression analyses described in the following sections were performed with generalized estimating equations (GEE) models with a nine-dependent correlation matrix. These models were used to take into account correlations between the repeated measurements of BMI within the same subject.

Both the overall association and age-specific associations between reported living room temperature and BMI were analyzed for children who lived in houses where the heating was used in the living room. These analyses were repeated for children who did not move during the entire 11 years study period, the non-movers. The latter was done because indoor temperature was reported at baseline and moving could contribute to a change in heating habits.

Like for living room temperature, both the overall association and age-specific associations between reported bedroom temperature and BMI were analyzed for children who used the heating in their bedroom. These analyses were also repeated for children who did not move during the study, the non-movers. In addition, the overall association and age-specific associations between reported bedroom temperature as dichotomous variable (“heating used” versus “heating not used”) and BMI were also analyzed.

For the subgroup of children with objectively measured bedroom temperatures available (with data loggers), the overall association between measured bedroom temperature and BMI was analyzed. In addition, for children in the subgroup with both a reported and a measured bedroom temperature available, the Pearson correlation coefficient was calculated between these temperatures.

GEE models were stratified by gender because the development of the BMI in childhood is different between boys and girls. Furthermore, GEE models were adjusted for the confounders birth weight, maternal education level (low, intermediate, high), maternal overweight before pregnancy (BMI < 25 kg/m^2^ and BMI ≥ 25 kg/m^2^), indoor smoking (yes/no) and breastfeeding (yes/no). Indoor smoking and breastfeeding were used as lifestyle indicators. Consideration of potential confounders was based on a difference greater than 10% in effect estimate between crude and adjusted model and/or theoretical considerations. Maternal overweight before pregnancy could be a confounder in the association between indoor temperature and BMI, but could also be an effect of indoor temperature. The latter means that maternal overweight before pregnancy would not be a determinant of indoor temperature and could not be a confounder in this case. For this reason two different adjusted models were analyzed; one model with adjustment for maternal overweight before pregnancy and one model without this adjustment.

### Multiple imputations

In the original dataset (n = 3 343), the percentage of missing values on the child’s BMI ranged from 6.4% at the age of 1 year to 36.4% at the age of 11 years. Since missing data from general population studies are unlikely to be missing completely at random (MCAR), our analyses might lead to biased results because of selection bias. For this reason, missing data on all variables were multiply imputed by using the Multivariate Imputation by Chained Equations (MICE) procedure [7] in the statistical program R 2.13.1 [8]. Results of the analysis on each of the 20 imputed datasets, of 3 963 children each, were combined using PROC MIANALYZE in SAS 9.2.

Analyses were first done with the original dataset consisting of children with complete data on living room and/or bedroom and confounders (n = 3 343). Analyses were repeated with 20 imputed datasets of 3 963 children each. In this article, results from the analyses of the imputed datasets were reported and compared with results from the analyses of the original dataset with complete data on living room and/or bedroom and confounders.

## Results

Table 1 shows the characteristics of the study population for the dataset with complete data on living room and/or bedroom and confounders, and for the imputed datasets. The imputed data show that almost all children (99.7%) lived in a house where the heating was used in the living room. The reported living room temperature ranged from 10°C to 30°C and the mean reported temperature for this room was 20.3°C. Of all children 68.1% slept in a bedroom where the heating was used. The reported bedroom temperature ranged from 4°C to 28°C with a mean temperature of 17.4°C.

**Table 1:** Characteristics of the study population

	**Baseline population**		**Baseline population**
	**Original data**		**Imputed data**
	**(n = 3 343)**		**(n = 3 963)**
	**%**		**%**
Gender			
*Female*	48.3		48.2
*Male*	51.7		51.9
Maternal education level			
*Low*	22.0		23.7
*Intermediate*	41.7		41.6
*High*	36.3		34.7
Indoor smoking			
*Yes*	27.3		28.7
*No*	72.7		71.3
Breastfeeding			
*Yes*	83.0		82.0
*No*	17.1		18.0
Maternal overweight before pregnancy			
*BMI < 25 kg/m*^*2*^	80.3		79.5
*BMI* ≥ *25 kg/m*^*2*^	19.7		20.5
	**Mean ± SD**	**N**	**Mean ± SD**
Birth weight (grams)	3 513 ± 541	3 343	3 507 ± 546
Living room temperature (°C)		3 333	
*Heating used*	20.2 ± 1.2	3 326	20.3 ± 1.3
*Heating not used*		7	
Bedroom temperature (°C)		3 240	
*Heating used*	17.4 ± 2.5	2 193	17.4 ± 2.5
*Heating not used*		1 047	
BMI z-score at 3 months	0.12 ± 1.05	2 837	0.11 ± 1.05
BMI z-score at 1 year	0.03 ± 1.01	3 129	0.04 ± 1.02
BMI z-score at 2 years	0.06 ± 1.07	2 991	0.04 ± 1.08
BMI z-score at 3 years	0.08 ± 1.06	2 727	0.08 ± 1.07
BMI z-score at 4 years	0.01 ± 1.03	2 428	0.02 ± 1.04
BMI z-score at 5 years	0.20 ± 1.10	2 191	0.21 ± 1.11
BMI z-score at 6 years	0.20 ± 1.04	2 325	0.21 ± 1.04
BMI z-score at 7 years	0.14 ± 1.05	2 411	0.14 ± 1.04
BMI z-score at 8 years	0.08 ± 1.03	2 367	0.07 ± 1.03
BMI z-score at 11 years	0.04 ± 1.05	2 125	0.01 ± 1.06

Tables 2, 3, 4 and 5 show the crude and adjusted associations between indoor temperature and BMI. Since the results of the two adjusted models (with and without maternal overweight before pregnancy) were hardly different, only the adjusted model including maternal overweight before pregnancy is shown in these tables.

**Table 2:** Associations between reported living room temperature and BMI (for heating used)

	**3 months**	**1 year**	**2 years**	**3 years**	**4 years**	**5 years**	**6 years**	**7 years**	**8 years**	**11 years**	**Overall**
	***β***^**1**^	***β***^**1**^	***β***^**1**^	***β***^**1**^	***β***^**1**^	***β***^**1**^	***β***^**1**^	***β***^**1**^	***β***^**1**^	***β***^**1**^	***β***^**1**^
	**(95% CI)**	**(95% CI)**	**(95% CI)**	**(95% CI)**	**(95% CI)**	**(95% CI)**	**(95% CI)**	**(95% CI)**	**(95% CI)**	**(95% CI)**	**(95% CI)**
**Crude**											
Girls	-0.02	-0.01	-0.03	-0.01	-0.02	-0.01	0.00	0.00	0.02	0.00	-0.01
	(-0.06,0.03)	(-0.05,0.03)	(-0.07,0.01)	(-0.05,0.03)	(-0.06,0.02)	(-0.05,0.04)	(-0.05,0.04)	(-0.04,0.04)	(-0.02,0.06)	(-0.04,0.04)	(-0.03,0.02)
Boys	-0.01	0.03	-0.02	0.01	0.01	0.03	0.02	0.02	0.02	0.01	0.01
	(-0.06,0.03)	(-0.01,0.07)	(-0.06,0.01)	(-0.03,0.05)	(-0.03,0.05)	(-0.01,0.08)	(-0.02,0.05)	(-0.02,0.05)	(-0.02,0.06)	(-0.03,0.05)	(-0.02,0.03)
**Adjusted**^**2**^											
Girls	-0.02	-0.01	-0.03	-0.01	-0.02	-0.01	-0.01	0.00	0.01	-0.01	-0.01
	(-0.06,0.02)	(-0.05,0.02)	(-0.07,0.01)	(-0.05,0.03)	(-0.07,0.02)	(-0.05,0.03)	(-0.05,0.04)	(-0.04,0.04)	(-0.03,0.05)	(-0.05,0.03)	(-0.04,0.02)
Boys	-0.01	0.03	-0.02	0.01	0.02	0.04	0.02	0.02	0.03	0.01	0.01
	(-0.05,0.03)	(-0.01,0.07)	(-0.06,0.02)	(-0.03,0.05)	(-0.02,0.05)	(-0.01,0.08)	(-0.01,0.06)	(-0.01,0.06)	(-0.01,0.07)	(-0.03,0.05)	(-0.01,0.04)

^1^Increase in BMI z-score per 1°C.

^2^Adjusted for maternal education level, birth weight, indoor smoking, breastfeeding, maternal overweight before pregnancy.

*p < 0.05.

**Table 3:** Associations between reported bedroom temperature and BMI (for heating used)

	**3 months**	**1 year**	**2 years**	**3 years**	**4 years**	**5 years**	**6 years**	**7 years**	**8 years**	**11 years**	**Overall**
	***β***^**1**^	***β***^**1**^	***β***^**1**^	***β***^**1**^	***β***^**1**^	***β***^**1**^	***β***^**1**^	***β***^**1**^	***β***^**1**^	***β***^**1**^	***β***^**1**^
	**(95% CI)**	**(95% CI)**	**(95% CI)**	**(95% CI)**	**(95% CI)**	**(95% CI)**	**(95% CI)**	**(95% CI)**	**(95% CI)**	**(95% CI)**	**(95% CI)**
**Crude**											
Girls	-0.01	0.01	0.00	0.00	0.01	0.01	0.00	0.01	0.02	0.00	0.00
	(-0.03,0.02)	(-0.02,0.03)	(-0.03,0.03)	(-0.03,0.02)	(-0.02,0.03)	(-0.02,0.04)	(-0.02,0.03)	(-0.02,0.03)	(-0.01,0.04)	(-0.02,0.03)	(-0.01,0.02)
Boys	-0.01	0.00	0.00	0.00	0.00	0.01	0.01	0.02	0.01	0.00	0.00
	(-0.03,0.02)	(-0.03,0.02)	(-0.03,0.02)	(-0.03,0.02)	(-0.02,0.02)	(-0.02,0.04)	(-0.02,0.03)	(0.00,0.04)	(-0.01,0.04)	(-0.03,0.03)	(-0.01,0.02)
**Adjusted**^**2**^											
Girls	0.00	0.01	0.00	0.00	0.01	0.02	0.00	0.01	0.02	0.01	0.01
	(-0.03,0.02)	(-0.01,0.03)	(-0.02,0.03)	(-0.02,0.03)	(-0.02,0.03)	(-0.01,0.04)	(-0.02,0.03)	(-0.02,0.03)	(-0.01,0.04)	(-0.02,0.03)	(-0.01,0.02)
Boys	-0.01	0.00	0.00	0.00	0.00	0.01	0.01	0.02	0.01	0.00	0.00
	(-0.03,0.02)	(-0.03,0.02)	(-0.03,0.02)	(-0.02,0.02)	(-0.02,0.02)	(-0.02,0.04)	(-0.02,0.03)	(0.00,0.04)	(-0.01,0.04)	(-0.02,0.03)	(-0.01,0.02)

^1^Increase in BMI z-score per 1°C.

^2^Adjusted for maternal education level, birth weight, indoor smoking, breastfeeding, maternal overweight before pregnancy.

*p < 0.05.

**Table 4:** **Association between the use of heating in the bedroom (yes versus no**^**1**^**) and BMI**

	**3 months**	**1 year**	**2 years**	**3 years**	**4 years**	**5 years**	**6 years**	**7 years**	**8 years**	**11 years**	**Overall**
	***β***^**2**^	***β***^**2**^	***β***^**2**^	***β***^**2**^	***β***^**2**^	***β***^**2**^	***β***^**2**^	***β***^**2**^	***β***^**2**^	***β***^**2**^	***β***^**2**^
	**(95% CI)**	**(95% CI)**	**(95% CI)**	**(95% CI)**	**(95% CI)**	**(95% CI)**	**(95% CI)**	**(95% CI)**	**(95% CI)**	**(95% CI)**	**(95% CI)**
**Crude**											
Girls	-0.02	0.01	-0.03	-0.06	-0.11	-0.16*	-0.17*	-0.14*	-0.08	-0.11	-0.07
	(-0.14,0.10)	(-0.10,0.12)	(-0.15,0.08)	(-0.19,0.06)	(-0.23,0.01)	(-0.29,-0.03)	(-0.29,-0.04)	(-0.25,-0.03)	(-0.19,0.03)	(-0.22,0.01)	(-0.15,0.01)
Boys	-0.08	0.13*	0.04	0.04	-0.01	0.03	0.01	0.00	0.08	0.07	0.03
	(-0.20,0.03)	(0.03,0.23)	(-0.06,0.14)	(-0.06,0.14)	(-0.12,0.09)	(-0.09,0.14)	(-0.10,0.12)	(-0.11,0.11)	(-0.02,0.18)	(-0.04,0.18)	(-0.04,0.10)
**Adjusted**^**3**^											
Girls	0.00	0.03	-0.01	-0.04	-0.09	-0.13*	-0.14*	-0.12*	-0.05	-0.08	-0.05
	(-0.12,0.11)	(-0.07,0.14)	(-0.12,0.11)	(-0.16,0.08)	(-0.20,0.03)	(-0.26,0.00)	(-0.26,-0.02)	(-0.22,-0.01)	(-0.16,0.05)	(-0.19,0.04)	(-0.12,0.03)
Boys	-0.07	0.14*	0.05	0.05	0.00	0.04	0.02	0.01	0.09	0.08	0.04
	(-0.18,0.04)	(0.04,0.24)	(-0.05,0.15)	(-0.05,0.15)	(-0.10,0.10)	(-0.07,0.15)	(-0.08,0.13)	(-0.09,0.12)	(-0.01,0.19)	(-0.02,0.19)	(-0.03,0.11)

^1^Used as reference group.

^2^Increase in BMI z-score per 1°C.

^3^Adjusted for maternal education level, birth weight, indoor smoking, breastfeeding, maternal overweight before pregnancy.

*p < 0.05.

**Table 5:** Overall association between measured bedroom temperature and BMI

		**Overall effect**
	***β***^**1**^	**(95% CI)**
**Crude**		
Girls	0.00	(-0.08,0.07)
Boys	0.32	(-0.07,0.71)
**Adjusted**^**2**^		
Girls	0.04	(-0.05,0.14)
Boys	-0.04	(-0.16,0.08)

^1^Increase in BMI z-score per 1°C.

^2^Adjusted for maternal education level, birth weight, indoor smoking, breastfeeding, maternal overweight before pregnancy.

*p < 0.05.

### Associations between reported living room temperature and BMI

No overall association and no age-specific associations were observed between living room temperature and BMI. After adjustment for confounders, all regression coefficients were non-significant and ranged from -0.03 to 0.04 BMI z-score per 1°C (Table 2).

#### Non-movers

For the non-movers (n = 1 293) also no overall association and no age-specific associations were found between living room temperature and BMI. After adjustment for confounders, all regression coefficients were non-significant and ranged from -0.04 to 0.05 BMI z-score per 1°C (data not shown).

### Associations between reported bedroom temperature and BMI

Neither an overall association nor age-specific associations were observed between bedroom temperature and BMI for children who used the heating in their bedroom. All regression coefficients were non-significant and ranged from -0.01 to 0.02 BMI z-score per 1°C after adjustment for confounders (Table 3). Analyses of the overall association between bedroom temperature as dichotomous variable (“heating used” versus “heating not used”) and BMI showed no significant differences between children who used the heating and children who did not use the heating in their bedroom. Analyses of age-specific associations for this dichotomous variable showed a significant difference for boys at the age of 1 year. Boys had a 0.14 higher BMI z-score compared to boys who did not use the heating in their bedroom. For girls significant differences were found at the ages of 5, 6 and 7 years. Girls had a respectively 0.13, 0.14 and 0.12 lower BMI z-score compared to girls who slept in a bedroom where the heating was not used (Table 4).

#### Non-movers

For the non-movers (n = 1 293) neither an overall association nor age-specific associations were observed between the continuous bedroom temperature variable and BMI with all non-significant regression coefficients ranging from -0.02 to 0.03 BMI z-score per 1°C after adjustment for confounders. For the non-movers neither an overall association nor age-specific associations were found between bedroom temperature as dichotomous variable and BMI. After adjustment for confounders, all regression coefficients were non-significant and ranged from -0.10 to 0.13 BMI z-score when the heating was used (data not shown).

### Associations for measured bedroom temperature and BMI

For children with both an objectively measured bedroom temperature and a reported bedroom temperature available, the objectively measured bedroom temperature ranged from 9.0°C to 21.4°C with a mean temperature of 17.5°C. This mean temperature was 0.1°C higher than the mean reported bedroom temperature for these children. Reported bedroom temperatures and measured bedroom temperatures were moderately positively correlated (*r* = 0.42, *p* = 0.001). No overall association was found between the objectively measured bedroom temperature and BMI. Table 5 shows that all regression coefficients were non-significant and ranged from -0.04 to 0.04 BMI z-score per 1°C, after adjustment for confounders.

### Comparison of the original dataset and the multiply imputed data

Analyses of the original dataset consisting of children with complete data on living room and/or bedroom and confounders (n = 3 343) showed very similar results compared to the analyses of the multiply imputed data. Also no overall association was found between indoor temperature (living room and bedroom) and BMI in the analyses of the original dataset. Analyses of the original data showed two more significant age-specific associations than the analyses of the imputed data. First, for reported living room temperature and BMI at the age of 5 years for boys (β *=* 0.09 BMI z-score per 1°C; data not shown). Second, for reported bedroom temperature, analyzed as dichotomous variable, at the age of 8 years for boys (β = 0.13 for “heating used”; data not shown). Analyses for the non-movers showed no overall association and no age-specific associations between indoor temperature and BMI in both the analyses of the original and imputed data.

## Discussion

Our study showed no evidence for an association between indoor temperature (living room and bedroom) and BMI in children between the age of 3 months and 11 years. We observed no associations between reported or measured indoor temperature and BMI. Whereas a few significant age-specific differences in BMI were found between children who used the heating in their bedroom and children who did not use the heating in their bedroom, no overall (over the entire age range) associations were observed between use of heating in the bedroom and BMI.

### Strengths and limitations

In contrast to experimental studies on the association between ambient temperature and energy expenditure, our study on the association between indoor temperature and BMI was conducted in a real life setting. We used a large population and prospective data with repeated measurements of BMI at 10 different ages, starting in the first year of life. We had data on living room as well as bedroom temperatures and for the bedrooms we had reported as well as measured data. Data on many potential covariates were available in the dataset. We used multiple imputation to make efficient use of the available data and to avoid bias due to potentially selective missing data. Furthermore, we conducted sensitivity analyses in the subgroup of families who lived in the same house throughout the 11 year study period.

This study also has some limitations. Data on living room and bedroom temperature and data on weight and height (used for calculating the outcome variable BMI) were parent-reported. In the PIAMA birth cohort study, the agreement between parent-reported and measured weight and height were assessed at the child’s age of 4 years [9] and 8 years [10]. On average, weight was underreported by 0.5 kg in children in the highest BMI quartile, but agreement in ranking of the children based on their BMI was very good. Both studies therefore concluded that the parent-reported data on weight and height can be used to study determinants of BMI in epidemiological studies. Furthermore, it is not likely that the underreporting of BMI depends on indoor temperature. To our knowledge, no research is available on the agreement between reported and measured indoor temperature. In our study, reported habitual bedroom temperatures were significantly moderately correlated with bedroom temperatures measured with data loggers during a 2-week period.

Living room and bedroom temperature were reported at baseline and we assumed that heating preferences remained stable during the years of the study. As we expected that heating habits might change when families move to another house, we conducted sensitivity analyses in the subgroup who never moved during the study period. Also in this subgroup, no associations were observed between indoor temperatures and BMI. Furthermore, there was no evidence for associations specifically at the earliest ages, i.e. the ages that were closest to the time of the exposure reporting. Lack of variation in temperatures is unlikely to be an explanation for the fact that no associations were found, because the range of both living room and bedroom temperature was quite large. Indoor temperatures at home were used as exposure variables. Children are also exposed to ambient temperatures outside their own home, for example in day care centers or at school, and we were not able to take these into account. However, children and especially young children spend a relatively large proportion of their time at home and a substantial proportion of their time in bed. Therefore, if there is an effect of indoor temperature on BMI, at least an association between bedroom temperature and BMI would be expected for this age group.

### Results of other studies and interpretation of the present study

Experimental studies in human subjects consistently found inverse associations between ambient temperature and energy expenditure under standardized conditions (i.e. for clothes, food intake and/or physical activity) [11-17]. An Italian observational population-based cohort study of 1597 Caucasian adults investigated the association between indoor temperature reported at baseline and incident obesity during 6 years of follow-up. Although adjustment for confounders considerably attenuated the risk for incident obesity, still a twofold increased risk for incident obesity was found in people reporting an indoor temperature higher than 20°C compared to people reporting an indoor temperature lower than or equal to 18°C [3]. In this study indoor temperature was reported at baseline, as was also the case in our study. Beside similarities between above-mentioned study and our study, there were also some differences. First, their study population consisted of adults, while our study population consisted of children. Since thermogenesis is known to differ between adults and children [18], results from studies in adults and studies in children cannot be easily compared. Furthermore, in the Italian study incident obesity during follow-up was used as outcome variable, while we used repeated measurements of BMI. In addition, in contrast to their study, we made a distinction between living room and bedroom temperature. Our own observational study did not provide evidence for the hypothesis that also under real life conditions ambient temperature affects energy balance and as a result influences BMI. Evidence from adult studies suggests that changes in eating behavior and clothing are likely to compensate at least partially for effects of ambient temperature on energy expenditure [14,19]. Mavrogianni et al. suggested in their review that the association between ambient temperature and energy expenditure could be weakened by behavioral factors in a real life setting [20]. In other words, families that keep their indoor temperatures relatively low might compensate by using more blankets and clothing to make their children comfortable. One of the earlier mentioned experimental studies did not standardize for clothes and bedding, as was done in all other experimental studies [14]. The subjects in this study wore more clothes and used warmer bedding at lower ambient temperatures. This resulted in a weaker association between ambient temperature and energy expenditure than in the experimental studies that did standardize for clothes and bedding. The mechanism suggested by Mavrogianni et al. could be an explanation for why lower ambient temperature is consistently associated with higher energy expenditure in experimental studies, but not with lower BMI in our real life study.

## Conclusion

This study in children did not support the hypothesized association between indoor temperature and BMI in a real life setting. Due to a lack of evidence for this hypothesis, we consider it premature to recommend turning down the thermostat as a measure to prevent overweight.

## Competing interests

The authors declare that they have no competing interests.

## Authors’ contributions

FRS was responsible for the statistical analysis, multiple imputation, interpretation of the results and the draft of the manuscript. MBMB and AHW contributed to the conception and design of the current study, interpretation of the results and critical revision of the manuscript. MS and UG gave statistical advice. AHW, MK, UG, GHK are principal investigators and senior researchers of the PIAMA study. They were responsible for the concept and the design of the PIAMA study and for data collection. All the authors were responsible for critical revision of the manuscript and they all read and approved the final manuscript.

## Pre-publication history

The pre-publication history for this paper can be accessed here:

http://www.biomedcentral.com/1471-2458/13/1119/prepub
